# Digital measurement and clinical significance of proximal femur in the older people of Inner Mongolia population, China

**DOI:** 10.1186/s12877-023-04254-w

**Published:** 2023-10-06

**Authors:** Li Jiawei, Zhang Kai, Wang Haiyan, Wu Chao, Zhang Yunfeng, Ding Liangjia, Liu Qinghua, Li Canran, Jin Feng, Li Xiaohe

**Affiliations:** 1 Department of Imaging, The Second Affiliated Hospital of Baotou Medical College, Baotou, 014030 Inner Mongolia Autonomous Region China; 2 Department of Anatomy, Inner Mongolia Medical University, Hohhot, 010000 Inner Mongolia Autonomous Region China; 3 Department of Orthopedics, The Second People’s Hospital of Ulanqab, Ulanqab, 012000 Inner Mongolia Autonomous Region China; 4 Department of Imaging, The Second Affiliated Hospital of Inner Mongolia Medical University, Hohhot, 010000 Inner Mongolia Autonomous Region China; 5 Department of Imaging, Affiliated Hospital of Inner Mongolia Medical University, Hohhot, 010000 Inner Mongolia Autonomous Region China

**Keywords:** Age, Gender, Inner Mongolia population, China, Morphometric measurement of proximal femur, Femoral prosthesis design

## Abstract

**Objective:**

This study aimed to measure the parameters of the proximal femur in the older people of Inner Mongolia, China and understand the influence of age and gender so as to provide guidance for the design and improvement of prosthesis for total hip arthroplasty.

**Methods:**

A total of 236 patients who underwent CT angiography of lower limbs in the Department of Imaging, Affiliated Hospital of Inner Mongolia Medical University of China were collected. They were divided into 4 groups according to age: < 60 (group A), 60–69 (group B), 70–79 (group C), and > 80 years (group D). Four anatomical parameters, including femoral head diameter (FHD), femoral neck-shaft angle (FNSA), femoral offset (FO), femoral neck anteversion (FNA), were measured by Mimics 21.0. Comparisons were made between age groups of the same gender and between genders in the same age group to analyze the correlation of the 4 parameters of proximal femur with age and gender. In addition, the results of this study were compared with previous studies.

**Results:**

There were no significant differences in FHD and FO between age groups, indicating no correlation with age. FNSA and FNA were no significantly different between group C and group D in the same gender, whereas there were significant differences between other age groups and were negatively correlated with age. There were significant differences in FHD and FO between genders in the same age group, with the males being larger than the females. FNSA and FNA were no significant differences between genders in the same age group.

**Conclusions:**

FNSA and FNA decrease with age. FHD and FO were larger in males than in females in all age groups. Age and gender should be considered in the design of prosthesis.

## Introduction

Total hip arthroplasty (THA) is an effective end-stage treatment of osteoarthritis, femoral head necrosis, femoral neck fracture, and other hip disorders [1], which occur mainly in the older people [2]. Fitting of the prosthesis to the proximal femur is the key to the success of THA and helps to restore hip function after surgery. Therefore, designing and selecting a prosthesis that fits the proximal femur is of great importance. The prosthesis is designed based on the anatomical characteristics of the implant site. Hence, it is of practical value to investigate the anatomical characteristics of the proximal femur. Numerous studies have been undertaken on the anatomical characteristics of the proximal femur in Europe and the United States, which argue that age and gender have an impact on the anatomy of the proximal femur [3–5]. There are differences in the anatomical morphology of the proximal femur between Chinese and Western population [6], but there are few reports on the anatomical characteristics of the proximal femur in the Inner Mongolia population, China for different age groups and genders. As China has become an aging society, the number of hip replacements will continue to increase in the future. Therefore, it is very important to investigate the anatomical characteristics of the proximal femur in the population of the region and design suitable prostheses. To this end, this study collected lower limb CT data of 236 Chinese people. A total of 4 anatomical parameters of the proximal femur, including femoral head diameter (FHD), femoral neck-shaft angle (FNSA), femoral offset (FO), and femoral neck anteversion (FNA), were measured and studied to provide information for the further improvement of prosthesis.

## Patients and methods

### Patients

Patients who underwent CT angiography of lower limbs in the Department of Imaging, Affiliated Hospital of Inner Mongolia Medical University from 2019 to 2021 were collected. All CT data were obtained by GE Lightspeed spiral CT with slice thickness of 0.625 mm, voltage of 120 kV, and current of 100 mA. This study was approved by the Ethics Committee of Inner Mongolia Medical University (YKD202201135).

Inclusion criteria: (1) aged > 30 years; and (2) scanning of the full length of the femur.

Exclusion criteria: (1) poor image quality that affects measurement; and (2) conditions that affect the shape or bone mass of the proximal femoral medullary canal, such as hip deformity, local bone defect, bone tumor, and previous history of hip surgery.

Grouping: A total of 236 samples were finally collected, including 121 males and 115 females, with an average age of 67.66 ± 13.65 years. All persons gave their informed consent prior to their inclusion in the study. The sample was divided into 4 groups according to age: < 60 (group A), 60–69 (group B), 70–79 (group C), and > 80 years (group D). Both femurs were measured for all patients, and the average was used for comparison.

### Measurements

Mimics 21.0 software was used to measure 4 anatomical morphological parameters of proximal femur, including FNSA, FNA, FO, and FHD. Comparisons were made between age groups of the same gender and between genders in the same age group to analyze the correlation of anatomical parameters with age and gender. Moreover, the results were compared with previous studies.

2.1 Standard coronal plane is the plane passing through the axes of the femoral neck and the proximal femoral shaft.
2.2 Transverse plane is the plane perpendicular to the longitudinal axis of the femur.
2.3 Femoral offset (FO) is the vertical distance from the center of rotation of the femoral head to the femoral shaft axis.
2.4 Femoral neck-shaft angle (FNSA) is the angle between the axes of the femoral neck and the femoral shaft.
2.5 Femoral neck anteversion (FNA) [7] is the angle between the projection line of the femoral neck axis at the transverse plane of distal femur and the line connecting the posterior poles of the medial and lateral femoral condyle
2.6 Femoral head diameter (FHD) is diameter of a best-fit circle around the femoral head

## Methods

### Measurement method

#### Establishment of the basal plane

**Fig. 1:**
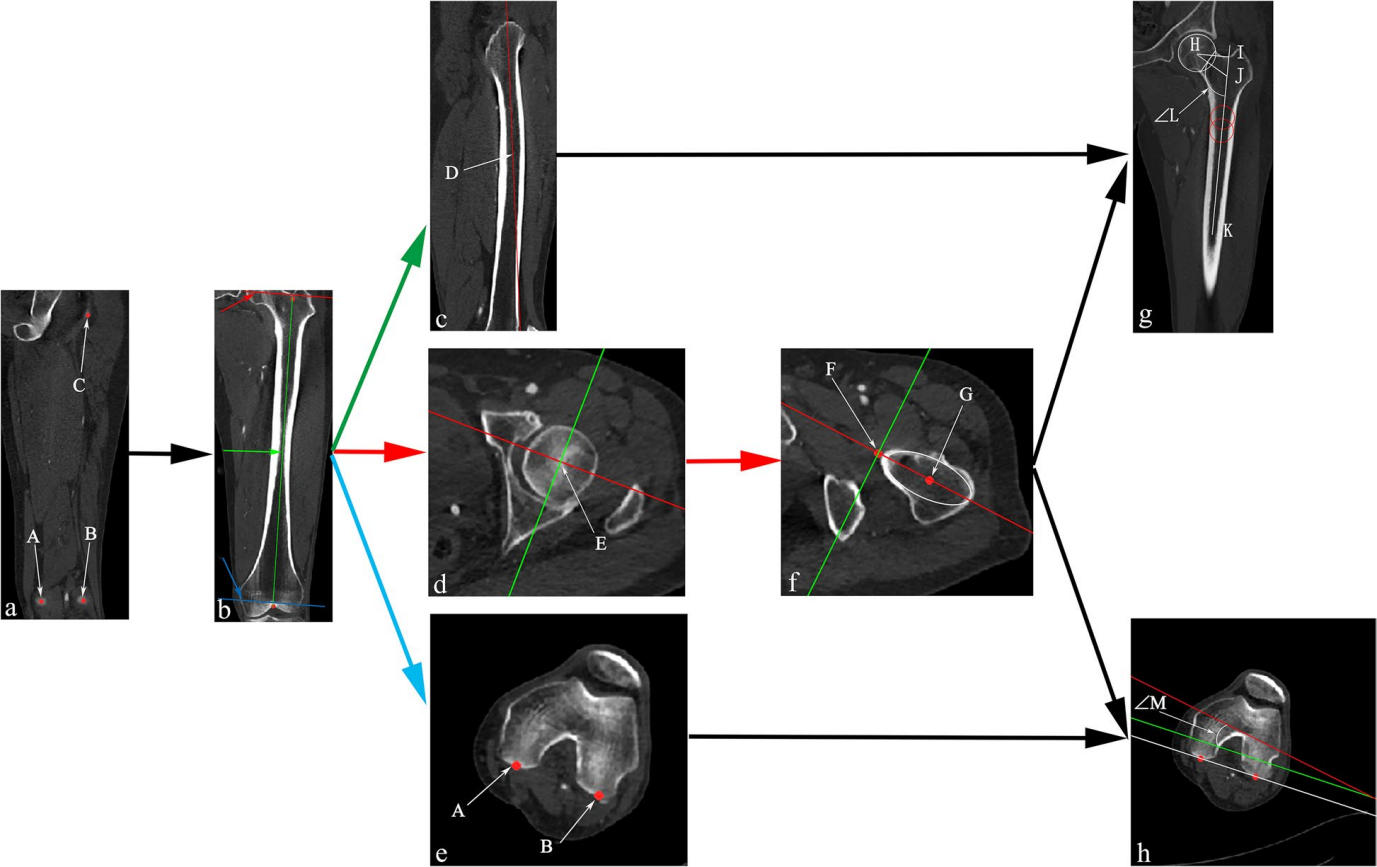
Measurement of FO, FNSA and FNA. **a** Establishment of the basal plane. The posterior pole of the medial and lateral femoral coondyle and the posterior pole of the greater trochanter were denoted as A, B and C, respectively. **b** This coronal plane was parallel to the basal plane, and the transverse plane and the sagittal plane were established in this coronal plane. **c** The Sagittal plane. The proximal femoral shaft axis was denoted as D in this plane. **d** The transverse plane. The center of the femoral head was denoted as E in this plane. **e** The transverse plane of distal femur. The posterior pole of the medial and lateral femoral condyle was denoted as A and B, respectively. **f** The transverse plane of the center of the base of the femoral neck. The femoral neck axis was denoted as FG. **g** the standard coronal plane. The FO and FNSA were measured in this plane, and were denoted as HI and ∠L, respectively. **h** The transverse plane of distal femur. FNA was measured in this plane, and was denoted as ∠M

Image data were imported into Mimics 21.0 (Materialise, Belgium). Next, the Interactive MPR tool under the Along Plane option in the VIEW option was selected to perform multi-planar reconstruction of thin-slice images. The posterior pole of the medial and lateral femoral condyle and the posterior pole of the greater trochanter were found to establish the basal plane, which passed through the posterior pole of the medial and lateral femoral condyle and the posterior pole of the greater trochanter (Fig. 1a).

#### Establishment of the transverse plane and femoral neck axis

A transverse plane was established perpendicular to the longitudinal axis of the femoral shaft in the coronal plane parallel to the basal plane (red and blue line in Fig. 1b). The center of the femoral head (denoted as E in Fig. 1d) was found in the transverse plane. The center of the base of the femoral neck (denoted as G in Fig. 1f) was found straight below. FG in Fig. 1f is the femoral neck axis (F in Fig. 1f is the projection of the center of the femoral head in this plane).

#### Establishment of standard coronal plane

The sagittal plane (Fig. 1c) was established parallel to the longitudinal axis of the femoral shaft in the basal plane (green line in Fig. 1b). The proximal femoral shaft axis was found in this plane (denoted as D in Fig. 1c). The standard coronal plane (Fig. 1g) was then established using Fig. 1c and f, which is through the femoral neck axis and proximal femoral shaft axis.

#### Measurement of FNSA, FO and FNA

A circle was drawn at and 2 cm below the lower edge of the lesser trochanter, respectively. The edges of the circles were tangent to the lateral cortex of the femur. The centers of the circles were determined. The axis passing through the two centers is the proximal femoral shaft axis. ∠L in Fig. 1g is FNSA. The line segment HI is FO.

The femoral neck axis was determined. The posterior poles of the medial and lateral femur condyle were then identified at the transverse plane of distal femur (Fig. 1e). The angle between the projection line of the femoral neck axis at the distal femur (red line in Fig. 1h) and the line connecting the posterior poles of the medial and lateral femoral condyle is FNA (∠M in Fig. 1h).

#### Measurement of FHD

**Fig. 2:**
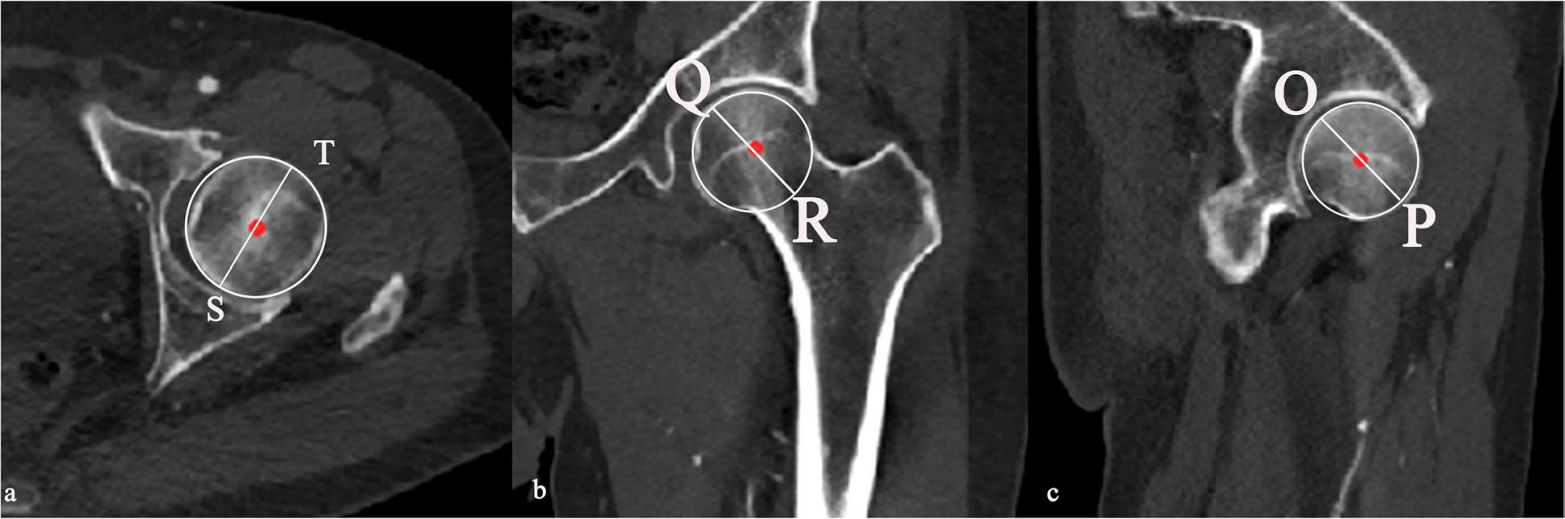
The measurement of FHD. The maximum diameter of the femoral head was identified and measured in the transverse (**a**), coronal (**b**), and the sagittal planes (**c**), respectively. The average of the three measurements was taken as the FHD value

The maximum diameter of the femur was identified and measured in the transverse, coronal, and sagittal planes, respectively (ST, QR and OP in Fig. 2a, b and c, receptively). The average of the three measurements, that is, (OP + QR + ST)/3, was taken as the FHD value.

### Statistical analysis

Data analysis was performed using SPSS 25.0. The data were expressed as mean ± standard deviation (x ± s) and tested for normality and homogeneity of variance.

#### Comparison of anatomical parameters of proximal femur between age groups of the same gender

Four groups of data were analyzed by one-way analysis of variance (ANOVA) and the least significant difference (LSD) test. *P* < 0.05 was considered statistically significant.

#### Correlation between anatomical parameters of proximal femur and age

Pearson correlation analysis was used. *P* < 0.05 was considered statistically significant.

#### Comparison of anatomical parameters of proximal femur between genders in the same age group

Independent samples t-test was used for intra-group comparison. *P* < 0.05 was considered statistically significant.

#### Comparison with previous studies

Summary independent samples t-test was used. *P* < 0.05 was considered statistically significant.

## Results

### Comparison of proximal femoral parameters between age groups of the same gender (Table 1)

**Table 1:** Comparison of proximal femur parameters among different age groups($\overline{x }$±s)

Proximal femur parameters of female	Group A	Group B	Group C	Group D	*F*	*P*1	*P*2	*P*3	*P*4	*P*5	*P*6
FHD (mm)	43.92 ± 2.66	43.69 ± 1.82	44.29 ± 1.55	43.89 ± 2.13	0.38	0.66	0.51	0.96	0.29	0.71	0.49
FO (mm)	35.62 ± 4.13	36.75 ± 3.31	35.89 ± 3.34	36.56 ± 3.33	0.68	0.21	0.78	0.31	0.38	0.83	0.50
FNSA (°)	128.27 ± 4.14	125.41 ± 3.31	122.71 ± 3.88	122.73 ± 3.10	**15.72**	**0.01**	**0.01**	**0.01**	**0.01**	**0.01**	0.98
FNA (°)	14.63 ± 2.83	13.26 ± 1.90	10.96 ± 2.62	11.16 ± 2.51	**14.54**	**0.03**	**0.01**	**0.01**	**0.01**	**0.01**	0.77
Proximal femur parameters of male	Group A	Group B	Group C	Group D	*F*	*P*1	*P*2	*P*3	*P*4	*P*5	*P*6
FHD (mm)	48.12 ± 2.29	47.59 ± 2.79	48.17 ± 2.13	48.29 ± 2.23	0.46	0.38	0.92	0.79	0.33	0.31	0.86
FO (mm)	38.49 ± 4.71	39.68 ± 4.13	39.04 ± 4.14	39.03 ± 3.81	0.41	0.27	0.59	0.65	0.55	0.59	0.99
FNSA (°)	129.31 ± 4.05	126.51 ± 3.39	123.64 ± 3.49	122.69 ± 3.02	**21.55**	**0.01**	**0.01**	**0.01**	**0.01**	**0.01**	0.33
FNA (°)	15.51 ± 2.79	13.00 ± 2.07	11.28 ± 1.95	10.20 ± 2.04	**31.47**	**0.01**	**0.01**	**0.01**	**0.01**	**0.01**	0.08

P1, groups A vs groups B; P2, groups A vs groups C; P3, groups A vs groups D; P4, groups B vs groups C; P5, groups B vs groups D; P6, groups C vs groups D

Four groups of data were analyzed by one-way analysis of variance (ANOVA) and the least significant difference (LSD) test. *P* < 0.05 was considered statistically significant

There were no significant differences in the FHD and FO between age groups of the same gender. There were significant differences in FNSA and FNA between all groups of same gender except between groups C and D.

### Comparison of proximal femoral parameters between genders in the same age group (Table 2)

**Table 2:** Comparison of proximal femur parameters between genders at the same age group($\overline{x }$±s)

Proximal femur parameters	Group A				Group B				Group C				Group D				
	male	female	t	*P*	male	female	t	*P*	male	female	t	*P*	male	female		t	*P*
FHD (mm)	48.12 ± 2.29	43.92 ± 2.66	6.93	**0.01**	47.59 ± 2.79	43.69 ± 1.82	6.41	**0.01**	48.17 ± 2.13	44.29 ± 1.55	8.97	**0.01**	48.29 ± 2.23		43.89 ± 2.13	7.05	**0.01**
FO (mm)	38.49 ± 4.71	35.62 ± 4.13	2.64	**0.01**	39.68 ± 4.13	36.75 ± 3.31	3.02	**0.01**	39.04 ± 4.14	35.89 ± 3.34	3.14	**0.01**	39.03 ± 3.81		36.56 ± 3.33	2.44	**0.02**
FNSA (°)	129.31 ± 4.05	128.27 ± 4.14	1.07	0.29	126.51 ± 3.39	125.41 ± 3.31	1.27	0.21	123.64 ± 3.49	122.71 ± 3.88	0.98	0.33	122.69 ± 3.02		122.73 ± 3.10	0.05	0.96
FNA (°)	15.51 ± 2.79	14.63 ± 2.83	1.24	0.22	13.00 ± 2.07	13.26 ± 1.90	0.49	0.63	11.28 ± 1.95	10.96 ± 2.62	0.56	0.58	10.20 ± 2.04		11.16 ± 2.51	1.43	0.16

Independent samples t-test was used for intra-group comparison. *P* < 0.05 was considered statistically significant

There were significant differences in FHD and FO between genders in the same age group, with the male averages higher than the female averages. There were no significant differences in FNA and FNSA between genders in each group.

### Correlation of proximal femoral parameters with age (Table 3, Fig. 3)

**Table 3:** Correlation analysis of femur proximal parameters and age in different genders

Proximal femur parameters	male		female	
	Correlation coefficient(r)	*P*	Correlation coefficient(r)	*P*
FHD (mm)	0.09	0.30	0.06	0.51
FO (mm)	0.01	0.99	0.09	0.33
FNSA (°)	**-0.47**	**0.01**	**-0.39**	**0.01**
FNA (°)	**-0.64**	**0.01**	**-0.49**	**0.01**

Pearson correlation analysis was used. *P* < 0.05 was considered statistically significant

**Fig. 3:**
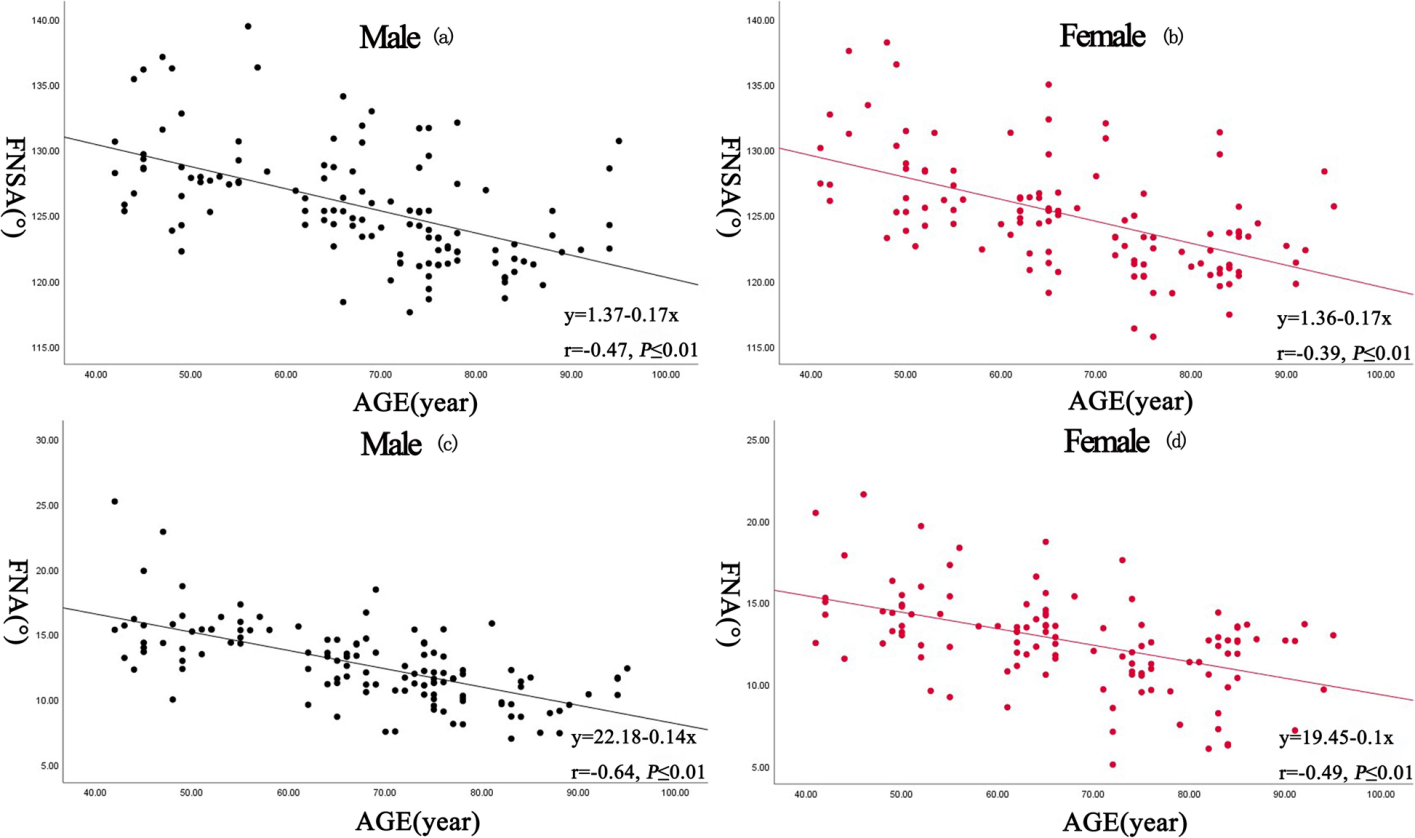
correlations between FNSA and age in Chinese males (a) and females (b). Significant positive correlations were noted between FNSA and age in both genders (*r* = 0.47, *P* ≤ 0.01, and y = 1.37–0.17 × for the males; *r* = 0.39, *P* ≤ 0.01, and y = 1.36–0.17 × for the females). Correlations between FNA and age in Chinese males (c) and females (d). Significant negative correlations were noted between FNA and age in both genders (*r* = 0.64, *P* ≤ 0.01, and y = 22.18–0.14 × for the males; *r* = 0.49, *P* ≤ 0.01, and y = 19.45–0.1 × for the females)

FNSA and FNA were negatively correlated with age. FHD and FO had no correlation with age.

## Discussion

Adequate fitting of the prosthesis to the femur after THA allows approximately normal load transfer and stress distribution in the proximal femur, thereby reducing stress shielding, which contributes to initial postoperative stability as well as long-term bone ingrowth and stability [8]. Related studies in European and American populations suggest that FNSA and FNA have a negative correlation with age and gender [4, 5], and that the mean FNA in females is greater than that in men, whereas the gender difference in FNSA is not clearly understood. There are certain differences in the proximal femur anatomy between Chinese and Western populations. It is still unknown whether the population in Inner Mongolia, China exhibits similar variations in the anatomy of the proximal femur. This study aims to not only understand the proximal femur anatomy in the Inner Mongolia population but also provide a scientific reference for designing prostheses that better suit the local population.

Current methods to measure proximal femoral parameters are X-ray, CT, and cadaver measurement. Among them, X-ray is commonly used, but it is easily affected by lower limb rotation. Rubin et al. [9] assessed the accuracy of X-ray and CT to measure the morphological parameters of the proximal femur, and found that CT had significantly less error than X-ray. In this study, the standard coronal plane reconstructed from multi-planar CT scans passed through the femoral neck axis and the proximal femoral shaft axis, eliminating the influence of femoral neck anteversion and thereby enabling more accurate measurements of FNSA, and FO.

The size of the femoral head prosthesis is related to joint dislocation and prosthesis interface wear after THA. It is believed that large-diameter femoral head prostheses significantly reduce the risk of postoperative dislocation, but they also increase the wear [10, 11]. Choosing an appropriate femoral head prosthesis helps reduce postoperative dislocation and wear of the prosthesis interface. Currently, a 36-mm femoral head prosthesis is recommended for uncemented THA in the United States, while a 32-mm femoral head prosthesis is recommended for cemented THA [10]. In this study, there was no statistically significant difference in FHD among the four age groups, whereas there was a significant difference between genders, with the male group being larger. Our results suggest that age does not affect FHD.

Appropriate FO and FNA reduce prosthesis failure and loosening [5, 12]. FO affects hip abduction strength and range of motion [13]. Too small FO in designing a femoral prosthesis will lead to increased instability and requires a long femoral neck for balance, thus resulting in unequal lengths of the lower limbs. In this study, there was no statistically significant difference in FO between different age groups of the same gender, whereas there was a significant difference between genders in the same age group. In a nutshell, FO is not related to age but related to gender. FNA varies greatly from birth to adulthood, and decreases by approximately 1.5° per year during childhood until growth is complete [14, 15]. FNA affects the biomechanics of the hip by changing its moment arm and the line of action of muscles around it. In THA, preoperative FNA affects intraoperative anteversion and orientation of the prosthesis [16, 17]. An appropriate FNA provides patients with satisfactory lower extremity function and prevents varus, valgus, impingement, and osteoarthritis [18]. Therefore, FNA should be evaluated before THA. In this study, we found that FNA was age-related, which is consistent with the findings of Pierrepont et al. [19]. However, there was no significant difference between genders in the same age group, which is different from the results of numerous Western studies [5]. This may be related to the small sample size of this study. FNSA affects the reconstruction of FO. A small FNSA of the prosthesis theoretically increases the FO [20], which in turn affects the abductor muscle strength and the range of motion of the hip. Charles et al. [21] and Bourne et al. [22] compared the FO reconstruction after THA with different FNSAs, and found that the FO reconstruction rate was 90.8% with a FNSA of 131°, whereas it was only 40.8% with a FNSA of 135°. Bachour et al. [23] preferred to use a femoral prosthesis with a FNSA close to the physiological one in THA. At present, commercially available femoral prostheses have a limited number of FNSAs, which are mostly 127° and 135°. In this study, we found that FNSA decreased with age, which is consistent with the findings of Fischer et al. [24]. Moreover, FNSA in the > 80 years group was approximately 122°, which was significantly different from 127° and 135°. It is recommended that for the older people, especially those above 80 years, the FNSA of the femoral prosthesis should be reduced accordingly to accommodate more older people. There is no sufficient evidence for gender differences in FNSA [4]. This study found no significant difference in FNSA between genders.

Finally, summary independent samples t-test was used to analyze the difference between this study and previous studies (Table 4). Since there are relatively few studies on these four parameters of the proximal femur for different age groups, we compared the four parameters in the total sample of different genders. FHD of male in this study had significant difference with Lee et al. [25] (*P* = *0.02*), and there was no significant difference with George et al. [26]. (*P* = *0.12*), whereas FHD of female in this study had significant difference with those reported by Lee et al. [25] (*P* < *0.05*), George et al. [26]. (*P* = *0.12*). FO measurements in this study were significantly larger than those reported by Takamatsu et al. [27] (*P* < *0.01*). FNA measurements in this study were significantly larger than those reported by Koerner et al. [28] (*P-White* < *0.05, P-African American* < *0.05, P-Hispanic* < *0.05*) except comparison with White population of female(*P-White* = *0.71*). FNSA measurements in this study were significantly smaller than those reported by Boese et al. [29] (*P* < *0.05*), Bagaria et al. [30] (*P* < *0.05*). The above comparisons reveal differences in the anatomical morphology of the proximal femur between the Inner Mongolia population and other populations, the study of the anatomical characteristics of the proximal femur is of great significance for the design of a more suitable prosthesis for the local population.

**Table 4:** Comparison of this study with previous studies ($\overline{x }$±s)

Variable	No. of patients	FHD		FO		FNA		FNSA	
		male	female	male	female	male	female	male	female
Present study	236	48.04 ± 2.34	43.93 ± 2.09	39.03 ± 4.23	36.22 ± 3.55	12.70 ± 3.00	12.63 ± 2.89	125.77 ± 4.38	124.97 ± 4.27
Lee et al.(malay)	169	47.40 ± 3.10	43.00 ± 2.00	-	-	-	-	-	-
George et al. (United States-B)	226	48.40 ± 2.60	42.20 ± 2.10	-	-	-	-	-	-
Takamatsu et al	98	-	-	36.00 ± 5.80	33.40 ± 4.90	-	-	-	-
Boese et al	400	-	-	-	-	-	-	129.60 ± 5.90	131.90 ± 6.80
Bagaria et al	211	-	-	-	-	-	-	127.70 ± 3.90	126.60 ± 4.80
Koerner et al.(White)	72	-	-	-	-	7.92 ± 9.67	12.91 ± 10.23	-	-
Koerner et al.(African American)	193	-	-	-	-	8.96 ± 9.96	8.19 ± 11.13	-	-
Koerner et al.(Hispanic)	63	-	-	-	-	8.74 ± 7.61	8.21 ± 9.35	-	-

Summary independent samples t-test was used. *P* < 0.05 was considered statistically significant

## Conclusions

Several conclusions of this papers have important clinical significance:

1. This study found that FNSA was negatively correlated with age, and FNSA gradually decreased with the increase of age in Inner Mongolia population. Especially for people over 70 years old, the average FNSA was about 122°, which was nearly 6° less than that of young people. The FNSA of Femoral prosthesis suitable for young and middle-aged people might not be suitable for people over 70 years old. Therefore, when designing of prostheses for people over 70 years old, the model of FNSA of femur prosthesis should be increased, and the FNSA of 122° might be suitable for most people in this age group.
2. This study found that FNA was negatively correlated with age, and FNA in people over 70 years old was close to 11°, which was reduced by 4° compared with young people in Inner Mongolia population. The influence of age on FNA should be considered when designing the prosthesis. For people over 70 years old, the FNA of the prosthesis should be relatively small, close to 11°.
3. There were significant differences in FHD and FO between genders, with the males being larger, and gender differences should be taken into account in the design of prosthesis.

## Data Availability

The data are not publicly available due to their containing information that could compromise the privacy of research participants. If someone wants to request the data from this study, please contact Li Jiawei.

## Abbreviations

FHD: Femoral Head Diameter
FNSA: Femoral Neck-shaft Angle
FO: Femoral Offset
FNA: Femoral Neck Anteversion
